# Supplementary material for: Prenatal Mercury Exposure and Infant Weight Trajectories in a UK Observational Birth Cohort
Source: Toxics. 2022 Dec 22;11(1):10. doi: 10.3390/toxics11010010 (PMC9864311; doi:10.3390/toxics11010010)
Supplement: Supplementary file 1 [file toxics-11-00010-s001.zip › toxics-2072956-supplementary.pdf]

**Table S1.** Missing data summary. The sample of children was selected based on the presence of maternal mercury and infant weight measurements, meaning that none were missing data for the exposure and outcome of interest. In total, 14% of data was missing, and the distribution of missing data is described in the table below. Pre-pregnancy BMI and alcohol consumption were the most missing (11% of cases missing these variables), followed by smoking habit and fish consumption (6%). Pre-pregnancy BMI and alcohol were measured in the same questionnaire, as were smoking and fish consumption, which suggests that the missingness is a result of participants not completing a particular questionnaire rather than not answering specific questions.

| **Maternal and Infant Characteristics in Complete Cases and Those with at Least One Variable Missing. Median (IQR) or Count (Percentage).** | | | |
|---|---|---|---|
| **Variable** | **Percent of Variable Missing** | **Complete Cases (*n* = 416)** | **Missing Data Cases** (*n* = 128) |
| Maternal age (years) | 3% | 29 (6) | 28 (6) |
| Parity | 4% | 1 (2) | 1 (2) |
| Education | 5% | | |
| None/CSE/ Vocational/O-level | | 248 (58%) | 58 (56%) |
| A-level/degree | | 168 (42%) | 44 (44%) |
| Pre-pregnancy BMI | 11% | 22.6 (4.1) | 22 (3.9) |
| **Smoking** | 6% | | |
| No | | 361 (87%) | 78 (86%) |
| Yes | | 55 (13%) | 16 (14%) |
| **Alcohol** | 11% | | |
| No | | 266 (64%) | 48 (68%) |
| Yes | | 150 36%) | 22 (32%) |
| **Oily fish consumption** | 6% | | |
| Never | | 157 (38%) | 42 (44%) |
| Once in 2 weeks | | 137 (33%) | 30 (31%) |
| 1+ times per week | | 122 (29%) | 23 (23%) |
| **White fish consumption** | 6% | | |
| Never | | 68 (16%) | 21 (20%) |
| Once in 2 weeks | | 165 (40%) | 40 (41%) |
| 1+ times per week | | 183 (44%) | 34(34%) |
| **Shellfish consumption** | 6% | | |
| Never | | 321 (77%) | 70 (72%) |
| Once in 2 weeks | | 78 (18%) | 22 (23%) |
| 1+ times per week | | 17 (4%) | <10 (<5%) |
| Mercury (µg/l) | 0% | 1.93 (1.1) | 1.81 (1.0) |
| Selenium (µg/l) | 0% | 110.6 (26.4) | 108.3 (23.5) |
| **Child sex** | 0% | | |
| Male | | 226 (54%) | 73 (57%) |
| Female | | 190 (46%) | 55 (43%) |

Categories with 5 or less individuals are supressed, and may include zero. The characteristics of mother and child do not appear to vary notably between those with complete variable data and those with at least one variable missing. Covariates with missing data were estimated using R and the *MICE* package, with 20 iterations of 9 imputations.

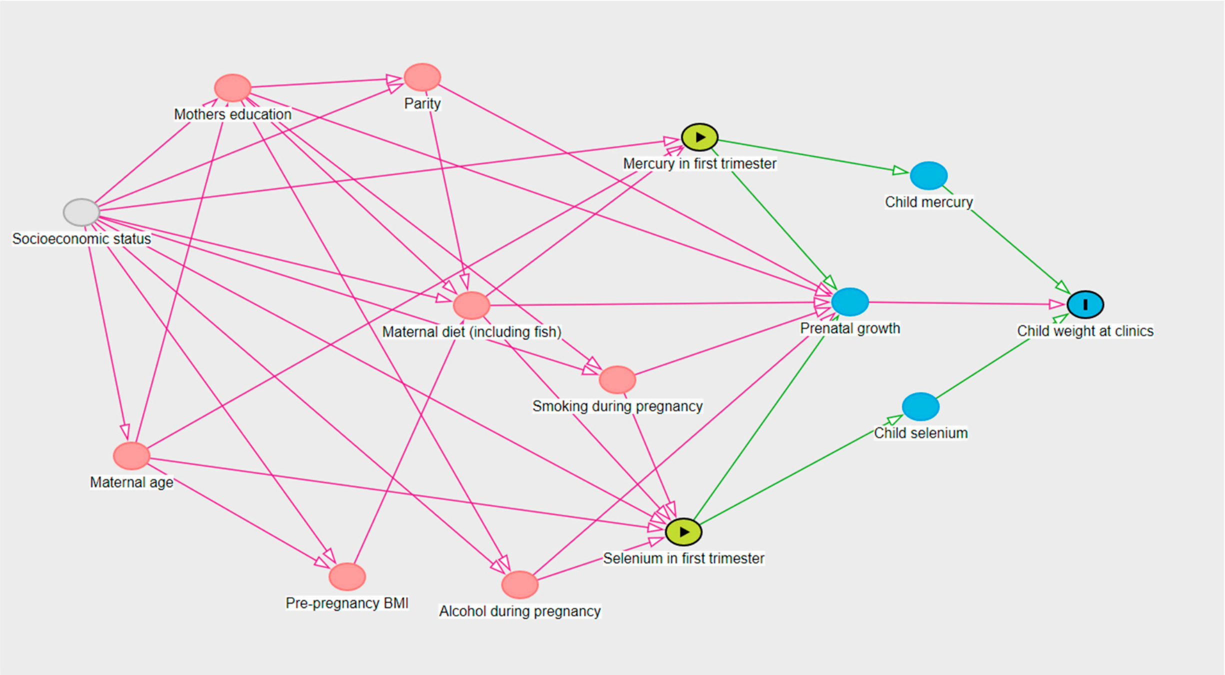


**Figure S1.** Directed acyclic graph of mercury, postnatal growth, and associated factors. Green circles represent the exposures of interest. Blue circles with "I" represent the outcome of interest. Red circles are ancestors of both the exposure and outcome, and therefore considered confounding variables. Gray circles are latent variables. The green arrows represent the causal pathway from exposure to outcome, and the red arrows represent confounding pathways.

**Table S2.** Full parameterisation of the mixed-effects model used to estimate change in postnatal growth from mercury and selenium.

| N = 544 | |
|---|---|
| **Outcome** | |
| Change in growth (kg) per month between 4 and 61 months | |
| **Variables** | |
| Mercury (1.048 µg/l) | |
| Selenium (26.66 µg/l) | |
| Maternal age | |
| Parity | |
| Pre-pregnancy BMI | |
| Maternal smoking (cigarettes per day) | |
| Maternal alcohol consumption (units/week) | |
| **Maternal education** | None/CSE |
| | Vocational |
| | O-level |
| | A-level |
| | Degree |
| **Oily fish consumption** | Never |
| | Once in 2 weeks |
| | 1-3 times per week |
| | 4+ times per week |
| **White fish consumption** | Never |
| | Once in 2 weeks |

| | |
|---|---|
| | 1-3 times per week |
| | 4+ times per week |
| **Shellfish consumption** | Never |
| | Once in 2 weeks |
| | 1-3 times per week |
| | 4+ times per week |
| **Child age (linear splines)** | |
| Child age (4 to 6 months) | |
| Child age (6 to 30 months) | |
| Child age (30 to 61 months) | |

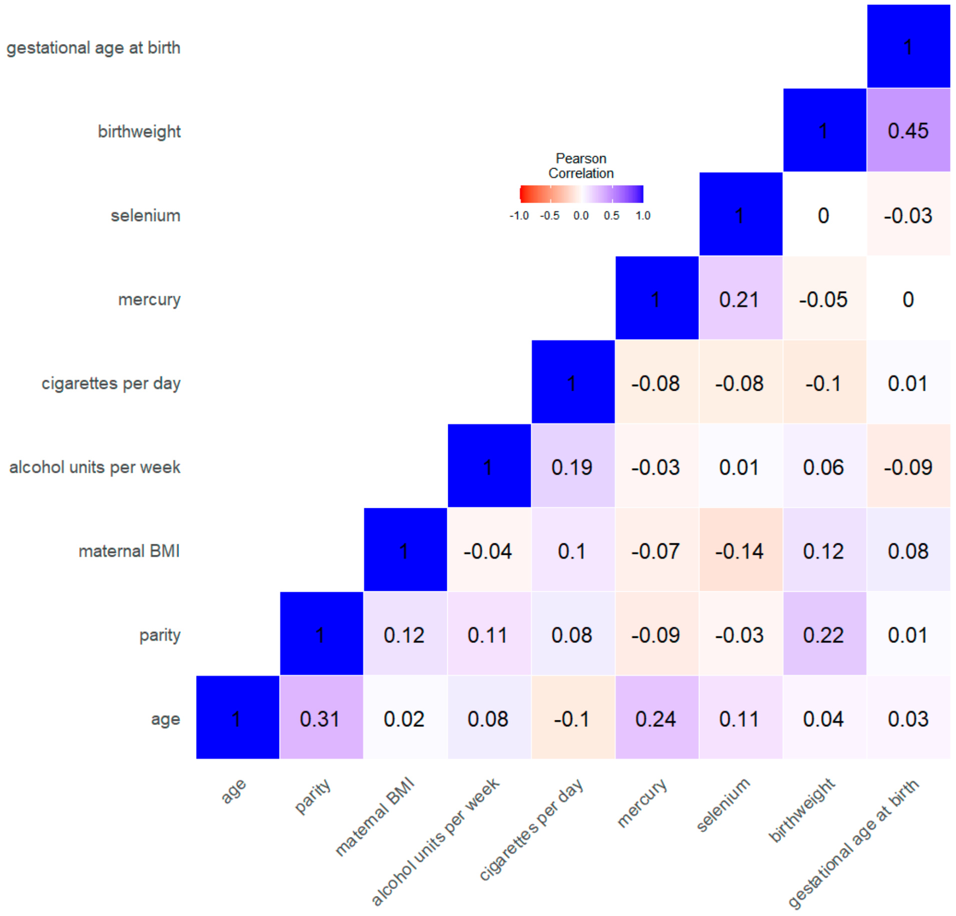


**(a).** Correlation heatmap of continuous variables.

Sex

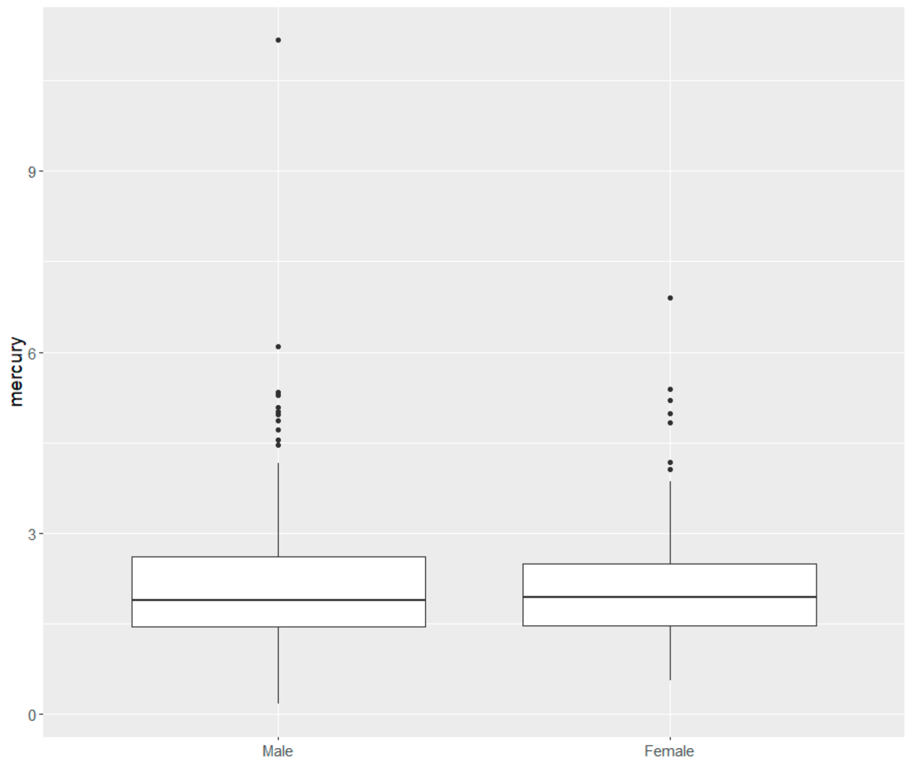

Shellfish consumption

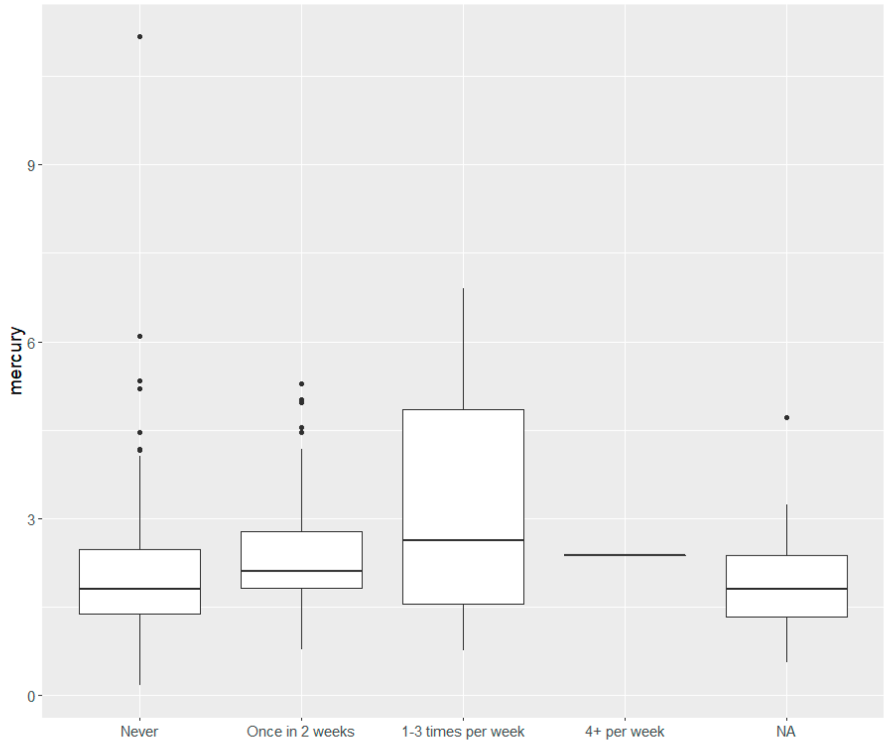


White fish consumption

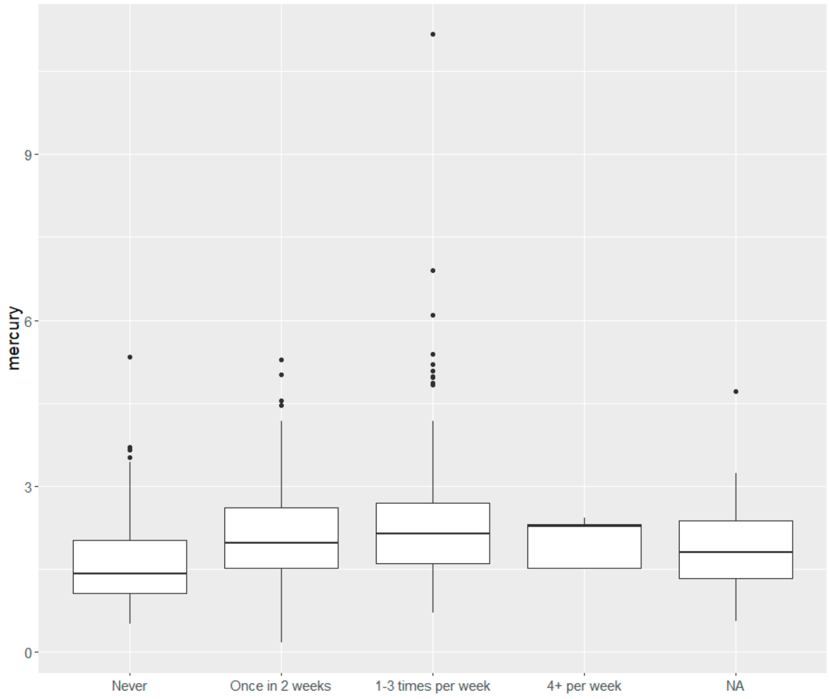


Oily fish consumption

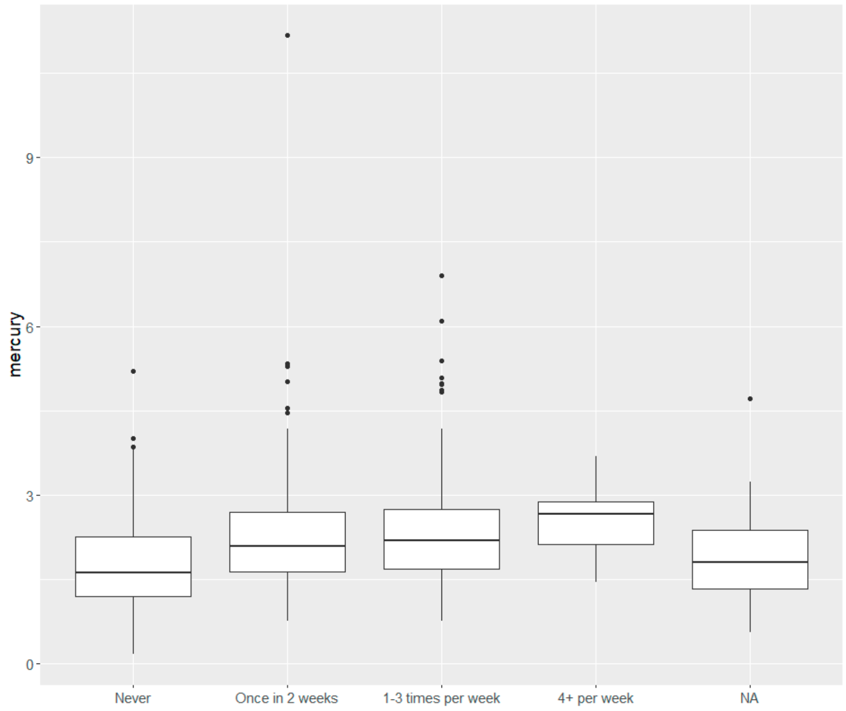


**(b)** Box plots of categorical variables and mercury.

Highest level of education

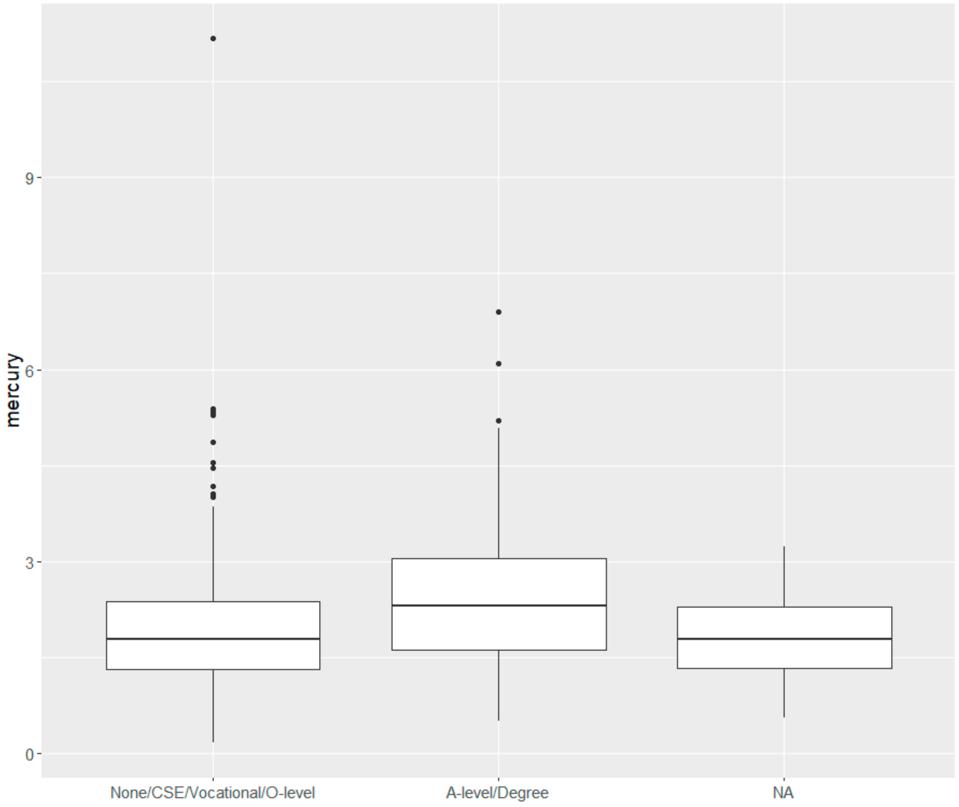

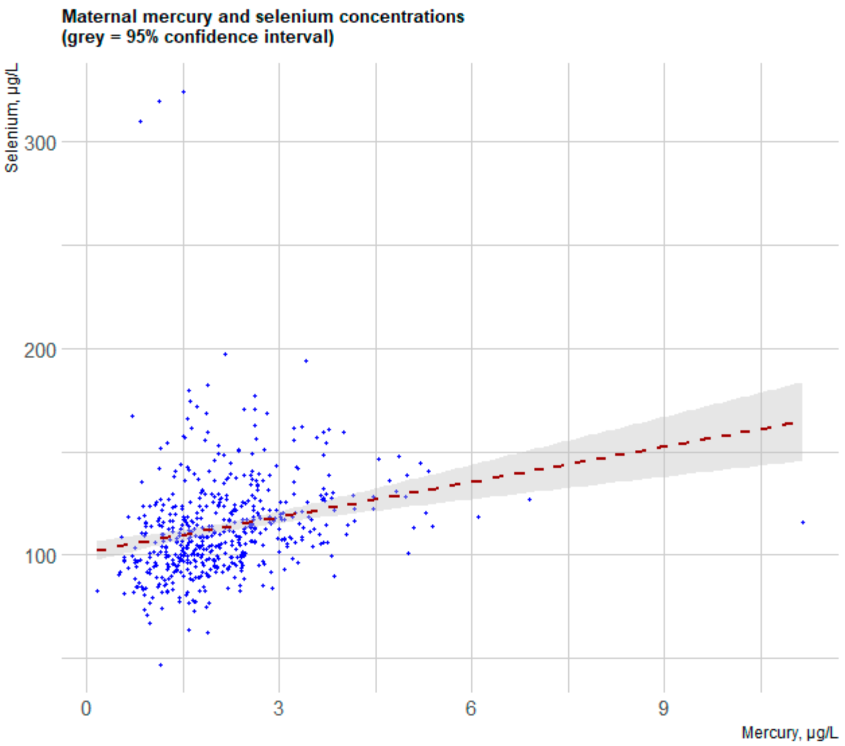


(**c**) Correlation and 95% confidence interval between maternal mercury and selenium whole blood measurements ($n$ = 544).

**Figure S2. a–c**. Comparisons between exposures and covariates.

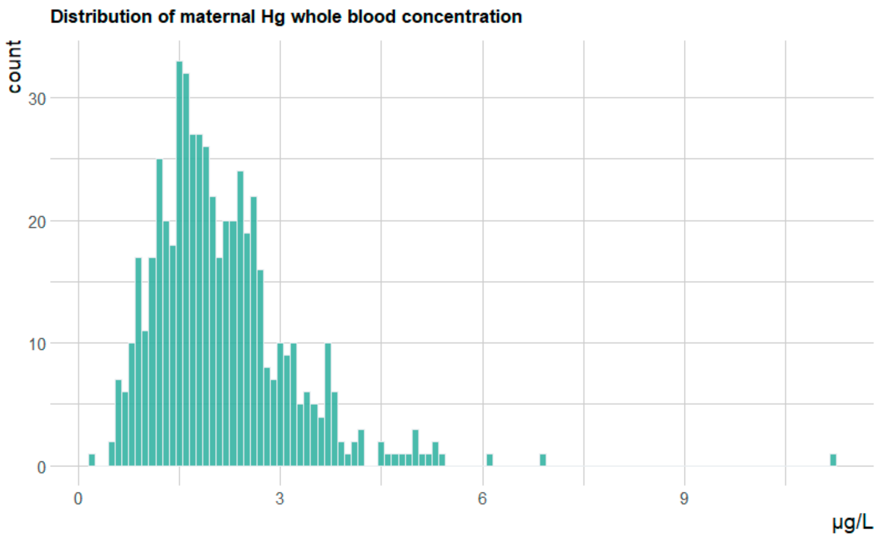


(**a**)

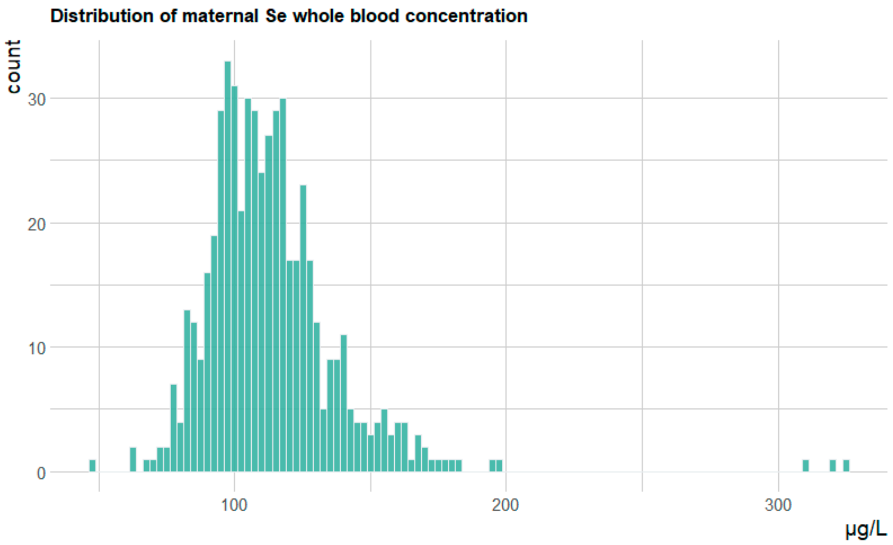


(**b**)

**Figure S3. a–b.** The distribution of mercury and selenium concentrations in maternal blood samples ($n$ = 544).
